# Intraoperative revision rates due to three-dimensional imaging in orthopedic trauma surgery: results of a case series of 4721 patients

**DOI:** 10.1007/s00068-022-02083-x

**Published:** 2022-09-01

**Authors:** Holger Keil, Nils Beisemann, Benedict Swartman, Marc Schnetzke, Sven Yves Vetter, Paul Alfred Grützner, Jochen Franke

**Affiliations:** 1 Department of Trauma and Orthopedic Surgery, Friedrich-Alexander-Universität Erlangen-Nürnberg, University Hospital Erlangen, Krankenhaus-street. 12, 91054 Erlangen, Germany; 2 BG Trauma Center Ludwigshafen at Ruprecht-Karls-Universität Heidelberg, Ludwig-Guttmann-Street. 13, 67071 Ludwigshafen, Germany

**Keywords:** 3D imaging, Fractures, Osteosynthesis, Intraoperative imaging

## Abstract

**Purpose:**

Intraoperative 3D imaging has become a valued tool in assessing the quality of reduction and implant placement in orthopedic trauma surgery. In our institution, 3D imaging is used routinely since 2001. To evaluate the intraoperative findings and consequences of this technique, intraoperative revision rates in cases with 3D imaging were analyzed.

**Methods:**

All operative procedures carried out with intraoperative 3D imaging between August 2001 and December 2016 were included. The scans were assessed intraoperatively and documented thereafter. In case of malreduction or misplaced implants, an immediate revision was performed. The number of scans per case as well as the findings and consequences drawn regarding the anatomical region were analyzed.

**Results:**

4721 cases with 7201 3D scans were included in this study. The most common anatomical regions were the ankle (22.3%), the calcaneus (14.8%) and the tibial head (9.5%).

In 19.1% of all cases, an intraoperative revision was performed. The highest revision rates were found with 36.0% in calcaneal fractures, 24.8% in fractures of the tibial plateau, 22.3% in injuries of the ankle.

In 52.0% of revisions, the reduction was improved regarding intra-articular steps or joint congruency. In 30.5% an implant was corrected.

**Conclusion:**

Intraoperative revision due to results of 3D imaging was performed in almost one-fifth of cases. This illustrates the improved possibilities to detect malreduction and implant misplacements intraoperatively and thus the abilities to improve surgical outcome.

**Level of evidence:**

III.

## Introduction

Anatomical reduction of fractures involving articular surfaces is essential for a good clinical outcome regarding function, joint preservation and pain levels postoperatively [1]. As well, it is crucial to avoid intra-articular placement of implants that can harm cartilage and adjacent structures.

**Fig. 1:**
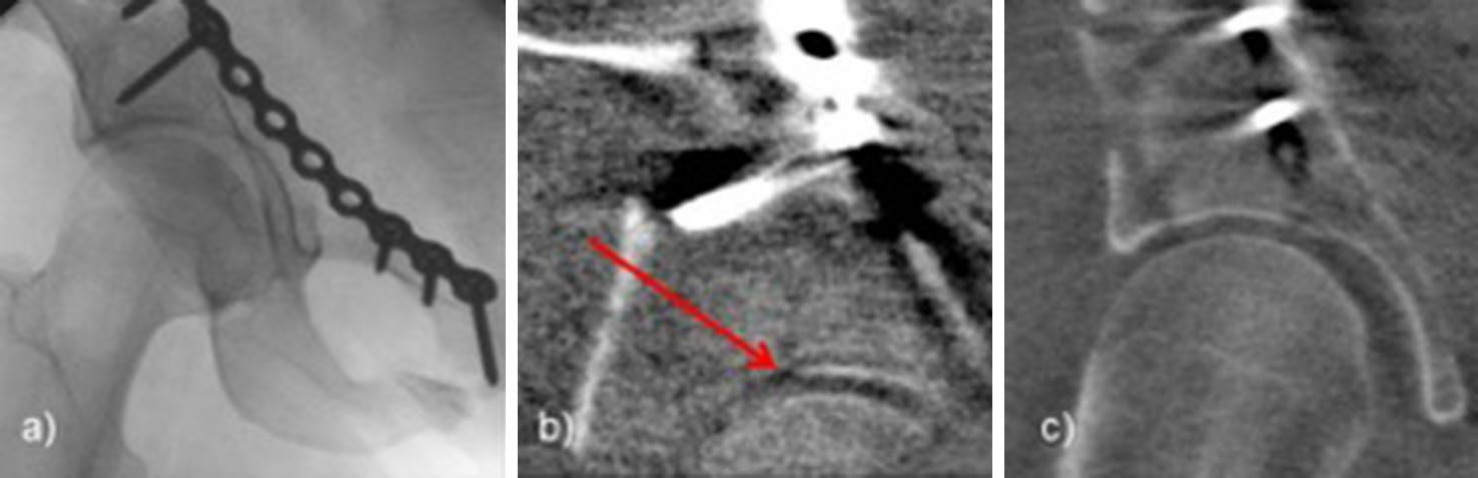
Male Patient, 49 y/o, Two-Column-Fracture of the right acetabulum. In the fluoroscopic ap view, the joint seems well reduced (**a**). In the 3D-Scan, an intraarticular step in the weight-bearing zone is revealed (**b**, arrow). After re-reduction, no step is left (**c**)

The standard of care is the intraoperative assessment of reduction and implant placement with intraoperative fluoroscopy. This allows 2D images to be acquired at any desired angle. With the help of these, sufficient evaluation of the operative result is possible in many cases. However, there are situations, when assessment with 2D imaging is not sufficient to exclude intra-articular implant placement or to ensure proper anatomical reduction of the articular surface [2–4]. This especially applies to concave or irregular joint surfaces like for example the acetabulum (see Fig. 1), the distal tibia or the calcaneus [5, 6]. Furthermore, the position of the fibula in the tibiofibular joint cannot be sufficiently assessed in 2D imaging to control joint congruency after the reduction and fixation of syndesmotic injuries [7, 8]. In these cases, where a definitive intra-operative assessment is not possible, the gold standard is to do a post-operative computed tomography (CT) to ensure sufficient documentation of the surgical result. In cases of malreduction or implant misplacement, the patient might need secondary revision surgery with all accompanying risks like anesthesiologic complications, wound infection and additional in-house days [9, 10].

To improve the possibilities of intra-operative assessment, three-dimensional (3D) imaging with mobile C-arms was introduced in 2001 [11]. These systems allow a CT-like three-dimensional visualization of the surgical area which allows for a far more precise evaluation of the result. Usually, these mobile devices can be used for 2D fluoroscopy as well, so there is no need for further equipment in the operation room.

Technically, the C-arm moves around the region of interest and automatically acquires several hundred dose-optimized 2D images. From these images, a 3D dataset is created by methods of filtered back projection or iterative reconstruction.

The main objective of this study was to use actual clinical experience from a large number of cases to identify critical anatomical regions with high revision rates as well as main findings in intraoperative 3D imaging over a long time span.

## Material and methods

From August 2001, intraoperative 3D imaging was used regularly in surgical procedures listed below. The institution is a large German Level I Trauma Center that is associated with a University Hospital.

From 2001 to 2006 the only device used was a Siemens Iso-C-3D. From 2006 two additional devices were added, each a Siemens Arcadis Orbic 3D (all devices: Siemens Healthcare GmbH, Erlangen, Germany). Besides several modifications regarding device handling and software improvements, there were no major differences regarding image acquisition and quality of image evaluation. The devices were available in all operating rooms.

Routinely, parameters and consequences drawn from the imaging were documented in a paper-based manner after the completion of the procedure by the surgeon. Items that were documented are listed in Table 1. To conduct this study, an electronic database that included all information from the paper-based documentation was created and used for further analysis.

**Table 1:** Datasheet of the 3D register

Item	Options/Comments
Consecutive number	
Patient age and gender	
Injured anatomical region	Acetabulum
	Ankle
	Calcaneus
	Distal radius
	Elbow
	Foot
	Anterior pelvic ring
	Posterior pelvic ring
	Cervical spine
	Lumbar spine
	Thoracic spine
	Talus
	Distal tibia
	Tibial plateau
	Other
Count of intraoperative 3D Scans	
Findings/Results of surgery	Free text
Intra-operative consequences of the 3D Scans	Yes
	No
Type of consequence	Improvement of reduction
	Replacement of an intra-articular screw
	Replacement of other screws

Indications for the use of intraoperative 3D imaging were.

- acquisition of the 3D data to be used in navigation
- ankle injuries with involvement of the syndesmosis
- fractures of the calcaneus, tibial plafond, tibial head, acetabulum and posterior pelvic ring
- fractures of the distal radius, elbow, talus, and tarsus depending on the degree of comminution and impression of the joint surface
- posterior pedicle screw placement of the thoracic spine or in cases with altered anatomical conditions

Contraindications for the use of intraoperative 3D imaging were.

- simple ankle fractures without syndesmotic lesion
- non- or minimally displaced fractures of the tibia that were treated with percutaneous screw osteosynthesis
- uncomplicated pedicle screw placement in the lumbar spine with good visibility in fluoroscopy
- glenoid fractures as these are hardly accessible with the imaging device
- severe obesity that made positioning of the C-arm impossible

**Fig. 2:**
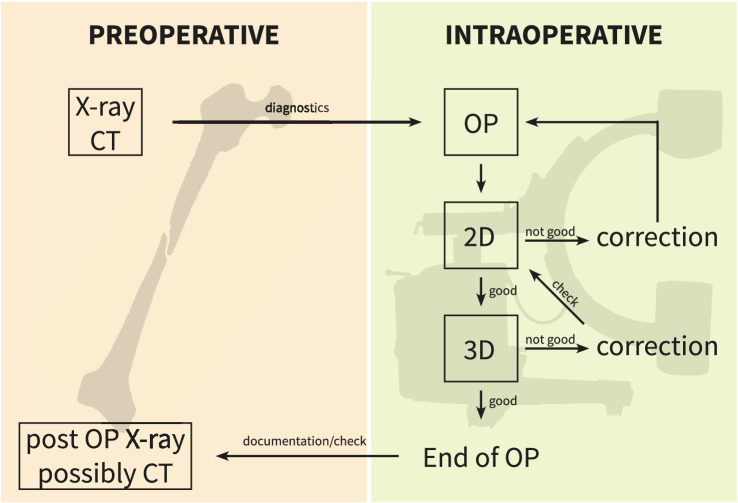
Workflow of intra-operative 3D imaging

Reduction maneuvers and implant placement were assessed by fluoroscopic imaging. At the point where the surgeon decided that the result is acceptable in 2D, the 3D scan was performed (see Fig. 2 for the workflow). The scan itself was performed by the OR staff. Editing and evaluation were done by the surgeon on the device. At this point, the decision was made whether revision was necessary or whether the procedure could be finished.

The surgeon cohort consisted of all fellow and attending surgeons that autonomously performed surgical procedures in the institution during the time mentioned above. In total, 40 surgeons were involved in the procedures. Regular demonstration and discussion of the cases were held to achieve a common level of understanding and training in intraoperative 3D imaging.

Criteria for intraoperative revision were:

Joints

- intra-articular implant positioning
- intra-articular fragments
- remaining intra-articular steps larger than 2 mm
- remaining intra-articular impressions larger than 2 mm
- malalignment of the adjacent joints, especially distal tibio-fibular joint

Spine

- intra-spinal screw or wire positioning
- affection of the neuroforamina
- penetration of anterior vertebral cortex
- extra-osseous course of screw or wire

For this retrospective medical record review, we analyzed the data regarding the injured anatomical region and the frequency of intra-operative revisions concerning these specific regions.

To identify possible anatomical regions with a high risk of the need for intraoperative revisions, logistic regression was calculated regarding the probability of the need of performing more than one scan depending on the anatomical region. Statistical analysis was done with SPSS 24 (IBM Corp., Armonk, USA).

Database creation and maintenance as well as data evaluation and analysis were done with Microsoft Excel 2016 (Microsoft Corp., Redmont, USA).

## Results

4721 cases with a total of 7201 evaluable 3D scans were included in this study.

The patients were treated between August 2001 and November 2016. The mean age was 46 ± 18.6 (Range: 13–97) years and there were 3027 male and 1694 female patients.

The average count of scans per case was 1.53 (Range: 1–17), the percentage of cases where there was more than one scan necessary was 37.2%.

The distribution of the different anatomic regions is shown in Table 2.

**Table 2:** Revision rates and measures taken in respect to the anatomical regions

Anatomical region	No. of cases	No. of 3D-scans	Average 3D-Scans per case	Percentage of all 3D-scans [%]
Ankle	1010	1607	1.59	22.3
Calcaneus	686	1065	1.55	14.8
Tibial plateau	485	686	1.41	9.5
Distal radius	405	479	1.18	6.7
Lumbar spine	355	538	1.52	7.4
Thoracic spine	329	585	1.78	8.1
Cervical spine	293	588	2.01	8.2
Distal tibia (pilon)	246	247	1.00	3.4
other	226	301	1.33	4.2
Acetabulum	205	325	1.59	4.5
Talus	153	238	1.56	3.3
Posterior pelvic ring	105	224	2.13	3.1
Foot	103	138	1.34	1.9
Anterior pelvic ring	84	136	1.62	1.9
Elbow	36	44	1.22	0.6

There were 384 scans that were acquired as a navigation image set for navigated procedures. These were excluded for further analysis as they did not show an operative result.

The total number of cases where an intraoperative revision due to findings in the 3D imaging was performed was 901. Thus, the overall revision rate of all anatomical regions was 19.1%.

Regarding the percentage of revisions regarding specific anatomical regions, the region with the highest revision rate was the calcaneus. 36.0% of all patients that underwent osteosynthesis there were revised after the 3D scan. The next frequent anatomical regions were the distal tibia with 24.8% and the ankle with 22.3%

**Fig. 3:**
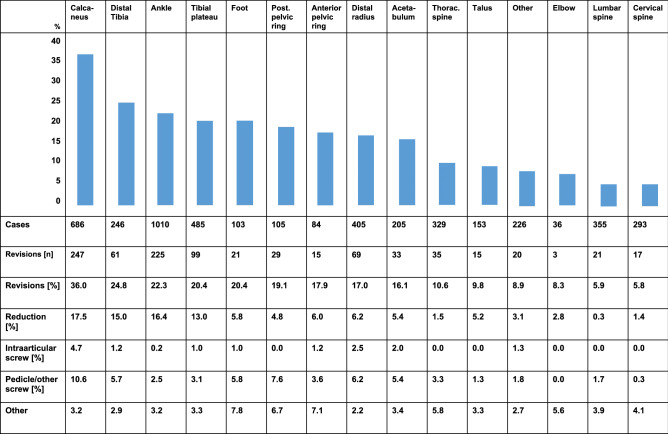
Representation of the revision rates and measures of the anatomical regions. The bars represent the revision rate of the respective region

Figure 3 shows a detailed analysis of the revision rates as well as the measures taken itemized to the anatomic regions.

In the regression analysis, Cohen’s (d) was calculated to 0.00, meaning a correlation between the anatomical region and the probability of receiving more than one scan could not be shown.

## Discussion

The results show a high percentage of findings in the intraoperative 3D images that led the surgeon to do an intraoperative revision of the osteosynthesis. Intraoperative revision rates as a direct consequence of the imaging were one-fifth averaged across all anatomical regions. These numbers are consistent with many of the data available in the literature regarding 3D imaging and the resulting consequences [6, 12]. It is well noticeable that the threshold for an intraoperative revision can be assumed to be lower than to indicate a (secondary) revision surgery from postoperative CT findings. Although the data in the current study does not allow for an analysis of the frequency of secondary revisions, it seems reasonable to suspect that this rate is lowered due to improved intraoperative assessment.

As illustrated above, 3D imaging is performed usually when fracture reduction and implant positioning are considered satisfactory in 2D imaging, so these values of intraoperative revision observed in this study show that there are various situations in which complete assessment of reduction and implant placement is not sufficiently possible in fluoroscopy. Beerekamp et al. described a change in the surgeon’s behavior in regard to how one relies on 2D or 3D imaging. In their study, they could show that if 3D is routinely used for calcaneal surgery, intraoperative revisions due to 2D findings decrease [13]. They did not examine the 2D radiation time, but from their own experience, it can be assumed that 2D fluoroscopy time decreases as the need for dynamic imaging and elaborate maneuvers often vanishes with the option of 3D.

3D imaging has variously been described as a valuable tool for assessing the quality of reduction and implant placement intraoperatively [14]. There are several studies regarding different anatomical regions that show a high accuracy regarding image quality—in many cases comparable to a CT scan—and a high value of the additional information retained by this modality in different anatomical regions [3, 12, 15–21].

In spine surgery, 3D imaging is used to assess the course of pedicle screws, especially in complex anatomical situations like scoliosis surgery or pedicle screw placement in the upper thoracic or cervical spine. Most authors find comparable results to our study with intraoperative revision rates between 5 and 10% [16, 22–25]. In addition, like in our collective, spinal procedures are more and more common to be navigated which was shown to further increase precision and reduce implant misplacement [26–28].

**Fig. 4:**
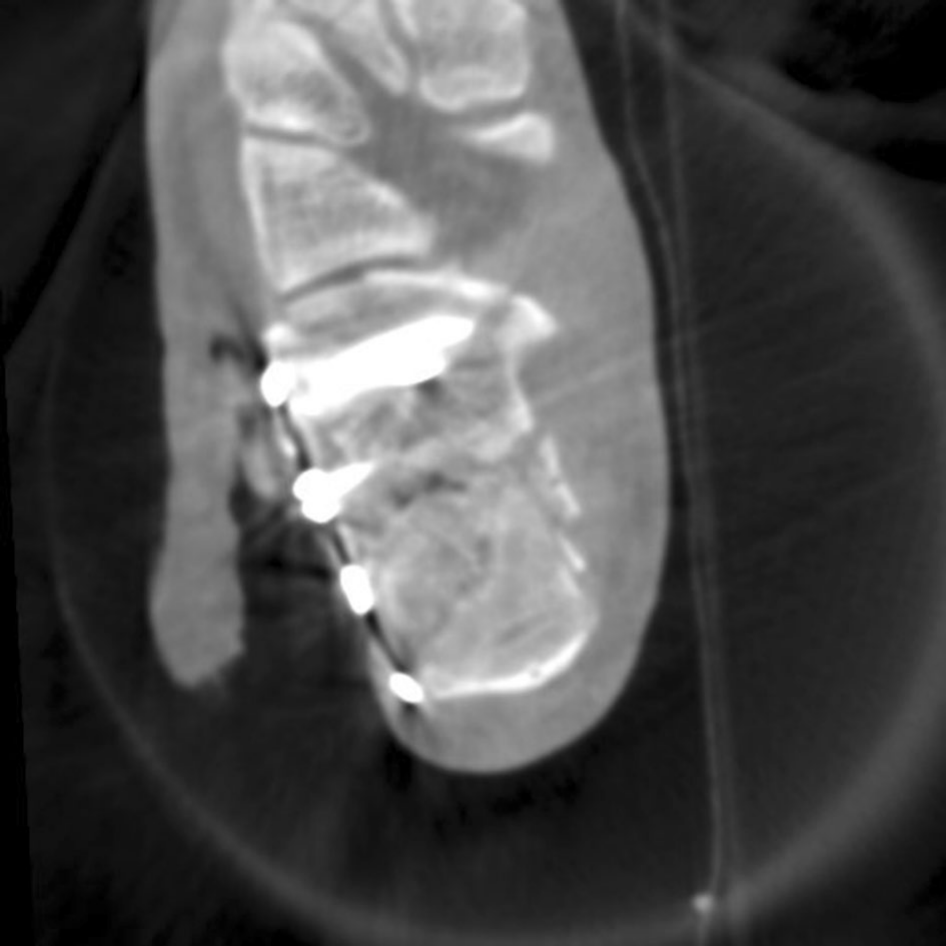
Male Patient, 32 y/o, Sanders Type III fracture of the calcaneus. Axial 3D scan of the calcaneus with positioned implants. Only in 3D, reconstruction of the calcaneo-cuboidal joint can be properly assessed, as well as the position of the implants

The calcaneus is a highly complex-shaped bone with articular surfaces to the talus as well as the other tarsal bones. These surfaces can hardly be completely assessed using 2D imaging due to superposing structures, so 3D imaging has been described as a very useful tool to evaluate the result of the surgery with a high rate of intraoperative revisions (see Fig. 4) [5, 13, 29]. This correlates to our findings with the highest rate of revisions. Additionally, this underlines that 3D imaging has its strength in overcoming the limitations of fluoroscopy especially in anatomic regions where complex anatomy cannot be evaluated in 3D.

In acetabular fractures, due to the concave shape of the acetabular dome and the anatomical location of the acetabulum with usually no possibility to directly evaluate the joint surface, 3D imaging has been described extremely helpful by many authors to evaluate reduction and implant placement [20, 30–32]. Revision rates have not been published by other groups; in our data the revision rates of the acetabulum are in the average of all anatomic regions. In a formerly published subgroup study, issues with the assessment in some conditions were present [33]. That illustrates the limits of intraoperative 3D imaging. New generations of devices will most likely help to improve image quality even under difficult conditions [34].

In ankle injuries with involvement of the syndesmosis, it is crucial to reduce the distal fibula correctly in the distal fibular-tibial notch. As of today, there is no validated method for the evaluation of this joint in 2D imaging, so the best possibility to assess this is an intra- or postoperative 3D- or CT imaging. We have observed an intra-operative revision rate of 22.28% in ankle injuries with involvement of the syndesmosis. Thus, we assume intra-operative 3D imaging as a valuable method to avoid revision surgery to correct the positioning of the fibula in the tibial notch if detected in postoperative CT. Our group as well as others have published comparable data regarding the intra-operative revision rate and the need for a tomographic view of the distal tibio-fibular joint [2, 7, 8, 12, 35, 36].

Not all anatomical regions are suitable for 3D assessment. It is very difficult to perform a 3D scan of the shoulder due to the extension of the thorax and the need to position the region of interest in the isocenter of the movement of the C-arm with the devices used in this study. Recently, newly designed machines (for example Ziehm RFD 3D, Brainlab Loop-X, Siemens Cios Spin 3D) with flat panel detectors instead of the formerly common image intensifiers allow for easier handling and more flexible patient positioning. Joint lines can be hard to assess when implants are present due to the generation of artifacts by the implants [37]. Obesity can lower the image quality as well. Nonetheless, usually even these images provide enough information to assess reduction quality. Also, there are more and more technical solutions coming up to reduce artifacts and improve image quality [38].

Another aspect that needs to be factored is the radiation exposure due to the 3D scan. The radiation dose emitted during a 3D scan is assumed to be comparable to that of a low-dose CT [39]. Usually, a postoperative CT scan is expendable if an intraoperative 3D scan was performed. We measured an average of 1.5 scans per patient, so there might be additional exposure to the patient. On the other hand, it seems reasonable to assume that fluoroscopy time is reduced when 3D is used in the procedure (see Beerekamp et al. [13]). In a previous study, dealing with the integration of a fan-beam-based intraoperative computed tomography device, the difference of radiation time between 2D and CT(3D)-based procedures was measured. There was a reduction of the average fluoroscopic radiation time of almost a factor of ten [40].

Nonetheless, be it additional or substitutive, the application of radiation always has to be justified. Although the data in the current study does not allow for outcome analysis, there have been subgroup analyses that show an improved clinical and radiological outcome for ankle and talus fractures that were operated with intraoperative 3D imaging [41, 42]. Regarding the exposure of the staff, there is an unambiguous situation, as the staff leaves the control area of the device during the scan. In combination with the above-mentioned reduction of fluoro time, this leads to a relevant reduction of radiation exposure. This is particularly true when navigated workflows are used [26, 43, 44].

This additional information comes for a price. A 3D-C-arm is more expensive than a 2D-device. The machine itself is often bulky, thus positioning of the C-arm might be more difficult to realize the standard views and the scan itself takes some time depending on the actual device used.

In a recently published meta-analysis of 31 studies, additional surgical time in cases performed with intra-operative 3D imaging was determined to 4.19 min [45]. Although not measured in all cases, this complies with our experience, that when draping is optimized for 3D movement and after some learning curve for the whole team, the additional time for preparing, performing and assessing the scan is around 5 min on average. In a study to assess first experiences with a novel intraoperative computed tomography device in acetabular surgery, additional time needed was measured to be 7 min after a learning time of eight cases [40].

Beerekamp et al. reported an extension of the operation time of 14 min in cases with 3D [13]. This might be due to a different device that needs separate draping for the scan movement or longer reconstruction times on the machine. If a relevant reduction failure or intra-articular screw is detected in the post-operative CT, time and resources consumed are much higher [46].

This study has several limitations. The register was created continuously and was analyzed retrospectively, so there is no comparability between patients that were operated with the help of intraoperative 3D imaging and those who were not. Due to the large cohort of surgeons that was involved in creating the database, individual learning processes and differences might have affected decisions. To minimize this factor, criteria for intraoperative revisions were defined as stated above and regular conferences ensured a comparable level of training of the individual surgeon. As the study was focusing on the radiological findings, patient-related endpoints like postoperative function or risk of osteoarthritis cannot be reported for the whole patient collective. As mentioned above, there have been analyses of subgroups that show a better clinical and radiological long-term outcome.

## Conclusions

In this analysis, a significant revision rate due to the results of intraoperative 3D imaging was observed. This shows a high value of this technique to improve the surgeon's capabilities to assess fracture reduction and implant placement. This applies especially to intra-articular fractures, ankle injuries affecting the syndesmosis as well as spinal procedures. These can be extremely difficult to assess in 2D imaging, so the supplementary information of 3D imaging helps to avoid the need for secondary revision surgery after evaluation in post-operative CT. In the strive for improvement of the reconstruction of complex fracture patterns, intraoperative 3D imaging creates new possibilities to identify malreduction and misplaced implants while the operative field is still accessible.

## Data Availability

Data is available on reasonable request.
